# Assessment of some hematologic markers of Systemic Inflammatory Response (SIR) as eliable biomarkers in detecting preeclampsia; a comparative cross-sectional study in Enugu, Nigeria

**DOI:** 10.4314/ahs.v25i3.8

**Published:** 2025-09

**Authors:** Theresa Ukamaka Nwagha, Helen Chioma Okoye, Joseph Tochukwu Enebe, Emmanuel Obiora Izuka, Kingsley Emeka Ekwuazi, Chiamaka Queenet Onyebuchukwu, Samuel Nnamdi Obi, Uchenna Ifeanyi Nwagha

**Affiliations:** 1 Department of Hematology and Immunology, College of Medicine, University of Nigeria/University of Nigeria Teaching Hospital, Ituku Ozalla Campus; 2 Department of Obstetrics and Gynecology, Enugu State University of Science and Technology College of Medicine/Teaching Hospital, Parklane, Enugu, Nigeria; 3 Department of Obstetrics and Gynecology, College of Medicine, University of Nigeria/University of Nigeria Teaching Hospital, Ituku Ozalla Campus; 4 Department of Human Physiology, College of Medicine, University of Nigeria, Enugu Campus; 5 Department of Reproductive Sciences, Institute of Maternal and Child Health, College of Medicine, University of Nigeria Ituku Ozalla Campus

**Keywords:** Preeclampsia, hematology markers, systemic inflammatory response, pregnancy, resource limited settings

## Abstract

**Background:**

Systemic inflammatory response (SIR) occurs in normal pregnancy and is exacerbated in pre-eclampsia (PE). Most markers of SIR are expensive to measure, require trained personnel, and thus are limited for routine application in resource-limited settings. Thus, we aim to determine the clinical utility of hematologic markers of systemic inflammatory response in Preeclampsia in a resource limited setting.

**Methods:**

A cross-sectional comparative study was conducted in Enugu from April to October 2021. Forty-five women with pre-eclampsia (PE) and 45 matched normotensive (NT) pregnant women were recruited. Hematological markers of systemic inflammatory response (red cell distribution width (RDW), neutrophil-lymphocyte ratio (NLR), platelet lymphocyte ratio (PLR), monocyte lymphocyte ratio (MLR), and platelet indices) were measured using the automated Mythic 22 Orphee hematology analyzer. Statistical analysis was by student T-test, receiver operating characteristics (ROC) curve, and Pearson correlation test. A P value of < 0.05 was considered significant.

**Results:**

The age difference between the groups was not significant (P = 0.76). Pregnant women with preeclampsia had significantly higher RDWS (P=0.02) and NLR (P <0.001) but a significantly lower PLR (P =0.04). The NLR had the largest area under the ROC curve (AUC 0.82, 95% CI (0.74-0.906, P <0.001), cut-off >2.56, sensitivity 71.1%; specificity 22.2%) followed by MLR (AUC 0.68, 95% CI (0.58-0.80, P =0.002) cut-off >0.18, sensitivity 64.4%; specificity: 28.9 %;)

**Conclusion:**

The NLR and MLR were good and affordable biomarkers of Systemic Inflammatory response (SIR) in Preeclampsia, thus possible alternative in resource-limited settings.

## Introduction

The systemic inflammatory response (SIR), unlike the local inflammatory response, is diffuse, involving all cells and proteins within the blood. It affects the inflammatory network in target organs and is not automatically due to antigenic stimulation.^1^ The key inflammatory cells involved in SIR include phagocytes (monocytes and granulocytes), dendritic cells, and NK cells. Endothelial cells are equally involved with a variety of pro-inflammatory cytokines, platelets, and a diverse group of humoral components, including complements and the clotting cascade.^2^

Pregnancy evokes a mild systemic inflammatory response, which varies from woman to woman. By the third trimester, it involves inflammatory leukocytes, endothelial activation, the acute phase response, and metabolic features of systemic inflammation.^2^ This systemic inflammatory response is exacerbated in preeclampsia(PE).^3^ Currently, the definition of PE as proposed by the International Society for the Study of Hypertension in Pregnancy (ISSHP) is new onset hypertension (systolic >140 mmHg or diastolic >90 mmHg) accompanied by one or more other features: proteinuria, other maternal organ dysfunction (including liver, kidney, neurological), or hematological involvement (thrombocytopenia), and/or uteroplacental dysfunction, such as fetal growth restriction and/or abnormal Doppler ultrasound findings of uteroplacental blood flow. 4

Globally, the burden of preeclampsia is high and is one of the most important causes of maternal and perinatal mortality and morbidity 5 and affects 2 to 8% of pregnancies worldwide.^5^ A recent maternal death and near-miss surveillance in Nigeria confirms that preeclampsia/eclampsia (PE/E) is now the leading direct cause of maternal mortality in health facilities; with PE/E and post-partum haemorrhage (PPH) accounting for 23.4% and 14.4% of severe maternal outcomes (SMO), respectively. 6

Interleukin-1(IL-1), TNF-alpha, IL-6, IL-8, and Interferon-gamma have been investigated as potential indicators of the SIR in normal pregnancy and preeclampsia.^7^ However, these studies reported that testing for these markers is expensive, laborious, requires trained personnel and has limited routine application. New evidence is emerging on the role of some hematological parameters as new markers of the systemic inflammatory response. These include the neutrophil-lymphocyte ratio (NLR), platelet-lymphocyte ratio (PLR), red cell distribution width (RDW).^8^,^9^ and monocyte to lymphocyte ratio (MLR).^10^ Others are platelet indices; plateletcrit (PCT), mean platelet volume (MPV), platelet distribution width (PDW), and platelet large cell ratio (PLCR).^11^

Some of these hematological markers of SIRS like RDW,^8^, NLR, 10 and PLR,^12^ hold great promise as predictors of preeclampsia. Some studies had also failed to show any correlation between MLR 9 and platelet indices^13^ and preeclampsia. These inconsistencies have made it difficult to determine which of these markers ideally predict and has diagnostic accuracy for diagnosing preeclampsia. The ongoing efforts to reduce maternal mortality rates in resource-limited settings like Nigeria and sub-Saharan Africa focus on early detection of preeclampsia and addressing gaps in the appropriate management of pre-eclampsia.^13^ There is then a need for simple, inexpensive, measurable, and widely used hematological markers of the systemic inflammatory response that can detect preeclampsia.

Thus, this study aims to determine the clinical value of these hematologic markers of systemic inflammatory response in detecting preeclampsia.

## Methods

**Study design:** This was a cross-sectional comparative study, conducted following the Strengthening the Reporting of Observational studies in Epidemiology (STROBE) statement.

**Setting:** It was conducted in Enugu, Southeast Nigeria between April, and October 2021. The booking clinics of 2 tertiary health facilities, the University of Nigeria Teaching Hospital (UNTH), Ituku-Ozalla, Enugu State University of Technology Teaching Hospital (ESUT-TH), and 3 secondary health care facilities, Mother of Christ Specialist Hospital (MCSH) Ogui Enugu, Balm of Gilead Specialist Hospital (BGSH) Maryland Enugu, and St Patrick Hospital (SPH) independence layout Enugu were conveniently chosen; based on the estimated number of clients assessing antenatal care in them and the presence of the required specialists in Obstetrics and Gynaecology. The average monthly ANC attendance for the hospitals were UNTH, 200, ESUT-TH, 700, MCSH, 80, BGSH, 200, and SPH, 200.

**Study Population:** Forty-five (45) pregnant women with preeclampsia were concurrently recruited using total population sampling, UNTH, 9/200, ESUT-TH, 22/700, MCSH, 6/80, BGSH, 8/200, and SPH, 0/200. We were not able to recruit any eligible patient from SPH due to logistic issues. Forty-five (45) matched normotensive pregnant controls were recruited by systematic random sampling. One out of every 30 eligible normal pregnant woman who met the inclusion criteria were recruited over a 6-month period. The sampling interval was based on the average estimated monthly ANC attendance, divided by the estimated sample size. The starting point was determined by simple random sampling using a lucky dip of YES or NO. Preeclampsia was defined as new onset hypertension (systolic >140 mmHg or diastolic >90 mmHg) accompanied by significant proteinuria (≥ 300mg protein in a 24-hr. urine sample) after 20 weeks gestation 4. The gestational age was calculated from the last menstrual period and confirmed using the first trimester ultrasound in the records. Where the first trimester was not available, early second trimester results were used. In additional obstetric ultrasound was also performed at recruitment. The women were at > 20 weeks of gestation and resided in Enugu and attended antenatal services at selected hospitals.

**Data Procedure:** The women were identified in the antennal clinic of the hospitals. During each ANC visit all pregnant wen were addressed, first by the matron in charge, then by any of the investigators present. All the procedures for the research were fully explained. All consenting women with preeclampsia who met the inclusion criteria were concurrently recruited. Systematic random sampling was used to recruit the control group.

### Inclusion and Exclusion criteria

All confirmed pregnant women with normotension or preeclampsia in Enugu whose gestational age > 20 weeks and who had seronegative HIV, HBV, and HCV syphilis were included in the study.

Exclusion criteria for the study were essential hypertension, seropositivity for HIV, HBsAg, HCV, syphilis, pre-existing conditions like diabetes, sickle cell disease, liver disease, renal disease, cardiovascular disease (CVD) and inflammatory disease (like arthritis, autoimmune disorders, asthma, chronic obstructive lung disease), acute malaria in pregnancy, diabetes in pregnancy and pregnancy complications (threatened miscarriage, antepartum haemorrhage.). Women on drugs that alter platelet function, and the body inflammatory response such as aspirin, clopidogrel, were also excluded from the study.

### Power and Sample Size Calculation

Statistical power analysis for a paired t-test was conducted using G*Power 3.1.9.7.^14^ The power calculation is based on four hematological markers of SIR in pregnancy induced hypertension (PIH) (MPV, PCT, PDW, and PLCR.). Thus, using a similar study by Manchanda et al.^15^ that yielded moderate to large effect sizes. We applied the Cohen's criteria^16^ to define the effect size for this study as moderate (Cohen's d=0.5). With a defined alpha level of 0.05, 95% power, and a two-tailed hypothesis, the estimated total sample size for this study is 42 participants. However, we recruited 45 participants within the study period of three months.

Ethical Clearance and Informed Consent Ethical clearance was obtained from UNTH HREC with the number NHRE/05/01/2008B-FWA00002458-IRB00002323. Written informed consent was also obtained from the patients.

### Study Variables

**Dependent Variables:** The primary outcome measures were some hematological markers of systemic inflammation; which include red cell distribution width (RDW), neutrophil-lymphocyte ratio (NLR), platelet lymphocyte ratio (PLR), monocyte lymphocyte ratio (MLR), platelet indices; Platetocrit (PCT), mean platelet volume (MPV), platelet distribution width (PDW), and platelet large cell ratio (PLCR).

**Independent Variable:** These were sociodemographic and clinical characteristics example, age, ethnicity, occupation, educational level, weight, height, BMI, parity, gestational age, blood pressure, and protein urine analysis.

**Data Collection:** Detailed obstetrics and pertinent medical history were obtained. Obstetrics, medical examination, and other relevant information were collected using a case record form.

Three ml of venous blood was collected aseptically and dispensed in an ethylene diamine tetraacetic acid (K3 EDTA) bottle for full blood count analysis which was evaluated in batches. A full blood count was performed using the Orphee Mythic 22 automated analyzer Switzerland:2012. All the samples were analysed in the hematology laboratory of UNTH, Enugu. White blood cell and platelet counts were quality controlled by viewing a peripheral blood film. The variables red cell distribution width (RDW), neutrophil-lymphocyte ratio (NLR), platelet lymphocyte ratio (PLR), monocyte lymphocyte ratio (MLR), platelet indices; platetocrit (PCT), mean platelet volume (MPV), platelet distribution width (PDW), and platelet large cell ratio (PLCR) were derived for both the test and control groups.

**Study Bias.** We ensure strict compliance with the sampling methods and the check list for the inclusion criteria. The automated machine was routinely calibrated and from time to time, manual methods were used for quality assurance.

**Study Size:** The sample size was calculated using the formula for comparison of independent means. A sample size of 45 was calculated for each group with 95% power to detect an increase of 5% at an alpha level of significance of 0.05 in a two-tailed analysis.

**Quantitative Variables:** All the quantitative variables were included in the analysis. There were no outliers.

**Statistical Methods:** Data were analyzed using the statistical package for social sciences (SPSS) computer software version 22.0 for windows (Chicago Illinois, USA). Quantitative variables were expressed as means SD, while categorical variables were presented as percentages, where applicable. The differences between the groups were analyzed using the independent student's t-test and Pearson's correlation analysis to determine the strength of the association. The receiver operating curve (ROC) and the area under the curve (AUC) were used to assess the sensitivity to these parameters to detect preeclampsia. All tests will be two-sided and statistical significance was at a probabilty value of < 0.05

## Results

Participants: Forty-five women with preeclampsia and 45 normotensive women were included in the data analysis. Descriptive Data

Sociodemographic characteristics of study participants: The age groups, education, occupation, and parity were not statistically significant between the groups. (P<0.05). Only the height difference and BMI between the groups was significant (P=0.03*) (See table 1).

**Table 1:** Socio-demographic and Pregnancy Characteristics of study participants, recruited from the antenatal clinic of the health care facilities in Enugu (Nigeria) from April, and October 2021 (N=90)

Variable		Group	P-value
	Test (N=45)	Control (N=45)	
Age Group (Years)			
**18 – 27**	16 (35.6)	12 (26.7)	
**28 – 37**	26 (57.8)	32 (71.1)	0.34
**>37**	3 (6.7)	1 (2.2)	
Mean age	29.89 ± 5.81	30.22 ± 4.57	0.76
			
**Highest Education Level**			
Secondary	16 (35.6)	10 (22.2)	0.25
Tertiary	29 (64.4)	35 (77.8)	
			
**Occupation**			
Civil servant	6 (13.3)	13 (28.9)	
Public Servant	6 (13.3)	5 (11.1)	
Business/Entrepreneur	20 (44.4)	11 (24.4)	0.24
Artisan	1 (2.2)	2 (4.4)	
Health Worker/Professional	3 (6.7)	6 (13.3)	
**Anthropometry**			
Systolic Blood Pressure (mmHg)	171.78 ± 22.49	106.44 ± 12.82	<0.001*
Diastolic Blood Pressure (mmHg)	112.67 ± 16.01	66.22 ± 10.51	<0.001*
Height (M)	1.62 ± 0.03	1.65 ± 0.07	0.03*
Weight (Kg)	90.11 ± 18.68	84.49 ± 16.76	0.14
Body Mass Index (Kg/M^2^)	33.85 ± 6.28	31.10 ± 6.45	0.04*
			
**Body Mass Index** **Classification**			
Normal (18.5 – 24.9 Kg/M^2^)	1 (2.2)	7 (15.6)	
Overweight (25 – 29.9 Kg/M^2^)	15 (33.3)	13 (28.9)	0.11
Obesity (≥ 30 Kg/M^2^)	29 (64.4)	25 (55.6)	
			
**Parity**			
None	12 (26.7)	20 (44.4)	
One	18 (40.0)	10 (22.2)	0.10
Two	7 (15.6)	11 (24.4)	
More than two	8 (17.8)	4 (8.9)	
**Gestation Week**			
< 37 Weeks	26 (57.8)	40 (88.9)	0.002*
>37 Weeks	19 (42.2)	5 (11.1)	

* Statistically Significant

### Outcome data

Hematology parameters in study Participants. Of the red cell parameters, only the difference in MCHC between the groups was statistically significant (P=0.002). The total red cell count, (P=0.42), hemoglobin (P=0.58), hematocrit, (P=0.11), mean cell volume (P= 0.36) and mean corpuscular hemoglobin concentration were not statistically significant (P=59). The differences between the groups in all parameters of white blood cells were statistically significant; total white cell count, lymphocytes, neutrophils (P < 0.001), monocytes (P=0.04), Eosinophils (P=.007). However the basophil count was not statistically significant (P= 0.11) (table 2)

**Table 2:** Mean values of hematological parameters by groups

	Test	Control	
WBC (10^3^/uL)	9.02 ± 3.52	5.52 ± 2.44	<0.001*
LYMPHOCYTES (%)	21.89 ± 7.67	35.14 ± 12.48	<0.001*
MONOCYTE (%)	4.56 ± 1.73	5.76 ± 3.16	0.04*
NEUTROPHIL (%)	72.14 ± 8.51	56.66 ± 15.39	<0.001*
EOSINOPHIL (%)	0.81 ± 0.78	1.82 ± 2.31	0.007*
BASOPHIL (%)	0.50 ± 0.20	0.63 ± 0.50	0.11
RED BLOOD CELL	3.68 ± 0.66	3.58 ± 0.42	0.42
(10^6^/uL)			
HGB (g/dL)	10.60 ± 1.87	10.43 ± 0.96	0.58
HCT (%)	32.03 ± 5.71	30.49 ± 3.00	0.11
MCV (um^3^)	87.50 ± 7.54	85.83 ± 9.51	0.36
MCH (pg)	29.03 ± 2.51	29.36 ± 3.15	0.59
MCHC (g/dL)	33.15 ± 1.19	34.27 ± 1.76	0.001*
PLT (10^3^/uL)	207.44 ± 57.66	216.73 ± 37.35	0.37

* Statistically Significant P=<0.05

### Hematological Markers of the Systemic Inflammatory Response (SIR) in study participants

The mean RDWS, NLR were significantly higher in the preeclampsia than normal pregnancy (P=0.02, and P <0.001) respectively. However, the PLR was significantly lower in preeclampsia (P = 0.04). The values of RDWC (P=0.54), MLR (P=0.60), PLT (P=0.37), MPV (P=0.46), PCT (P=0.32), PDW(P=0.18) and PLCR (P=0.59) were not statistically significant between the two groups. (table 3).

**Table 3:** Association between the Markers of SIR the Test and Control Groups (Student T-Test)

Variable	Group		t	P-value
**Blood Parameters**	Test	Control		
RDWC (%)	14.65 ± 1.33	14.49 ± 1.13	0.623	0.54
RDWS (um^3^)	46.78 ± 5.13	44.41 ± 4.52	2.328	0.02*
PLT (10^3^/uL)	207.44 ± 57.66	216.73 ± 37.35	-0.907	0.37
MPV (um^3^)	8.48 ± 0.97	8.64 ± 1.09	-0.748	0.46
PCT (%)	0.17 ± 0.41	4.43 ± 28.44	-1.003	0.32
PDW (%)	12.59 ± 1.77	13.06 ± 1.55	-1.356	0.18
PLCR (%)	15.22 ± 5.27	15.89 ± 6.39	-0.544	0.59
NLR	3.99 ± 2.41	1.92 ± 0.92	5.405	<0.001*
PLR	123.05 ± 45.63	156.10 ± 98.68	-2.040	0.04*
MLR	0.24 ± 0.12	0.21 ± 0.33	0.539	0.60

* Statistically Significant

### Correlation between SIR markers and systolic and diastolic blood pressure

The correlation analysis between the hematologic markers of SIR and systolic and diastolic blood pressure is shown in Tables 4. The Pearson correlation of the control group identifies a statistically significant but weak correlation between diastolic blood pressure and NLR (P=0.03) and PLR (P = 0.05), while for the pre-eclampsia group, there is a weak positive correlation between RDWC (%) and systolic blood pressure for the control group (P=0.04).

**Table 4:** Correlation between SIR Markers and Blood Pressure of study participants, recruited from the antenatal clinic of the health care facilities in Enugu (Nigeria) from April, and October 2021 (N=90)

Variable Markers	Systolic blood pressure				Diastolic blood pressure			
	Test (N=45)		Control (N=45)		Test (N=45)		Control (N=45)	
	r	p-value	r	p-value	r	p-value	r	p-value
RDWC (%)	-0.03	0.823	0.31	0.04*	0.18	0.237	0.12	0.44
RDWS (um^3^)	-0.06	0.682	-0.11	0.48	-0.01	0.947	0.15	0.33
PLT (10^3^/uL)	-0.18	0.232	0.13	0.40	-0.23	0.135	0.06	0.68
MPV (um^3^)	0.09	0.576	0.25	0.10	-0.03	0.840	0.17	0.26
PCT (%)	-0.17	0.278	0.16	0.29	-0.26	0.088	0.06	0.72
PDW (%)	-0.21	0.157	0.13	0.40	0.13	0.388	-0.07	0.66
PLCR (%)	0.01	0.969	0.18	0.24	-0.19	0.205	0.06	0.72
NLR	0.12	0.423	-0.24	0.11	0.22	0.147	-0.32	0.03*
PLR	-0.07	0.641	0.12	0.42	0.22	0.150	0.30	0.05*
MLR	-0.10	0.503	-0.10	0.54	0.16	0.285	0.06	0.69

* Statistically Significant

### ROC and AUC analysis of Hematological Markers for SIR to Detect Preeclampsia

The receiver operative curve (ROC) and the area under the curve (AUC) analysis for the SIR markers to detect preeclampsia are shown in Table 5. ROC analysis was plotted to determine optimal cutoff values for RDWS, RDWC, NLR, PLR, MLR and platelet indices Values for PLT, MPV, PCT, PDW, PLCR, and PLR below the cut-off would predict pre-eclampsia while values of RDWC, RDWS, NLR MLR greater than the cutoff would predict pre-eclampsia. NLR and MLR had good AUC, and sensitivity but poor specificity to distinguish between pre-eclampsia and normotensive pregnancy (NLR, cut-off >2.56, sensitivity 71.1%; specificity: 22.2%; AUC: 0.82, 95% CI (0.74-0.91), P<0.001; MLR, cut-off >0.18, sensitivity 64.4%; specificity: 28.9 %; AUC: 0.68, 95% CI (0.58-0.80), P= 0.002. In contrast, PLR, RDW. and platelet indices did not have diagnostic efficacy (AUC < 0.50) to distinguish normal pregnancy and PE.

**Table 5:** ROC/AUC Outcome of Hematological Markers of study participants, recruited from the antenatal clinic of the health care facilities in Enugu (Nigeria) from April, and October 2021 (N=90)

Marker	AUC (95%CI)	p-value	Cut-off	Sensitivity (%)	Specificity (%)
**RDWC (%)**	0.508 (0.387 – 0.629)	0.897	>14.65	40.0	48.9
**RDWS (um^3^)**	0.596 (0.478 – 0.715)	0.116	>47.05	37.8	31.1
**PLT (10^3^/uL)**	0.555 (0.434 – 0.676)	0.366	<191.50	40.0	24.4
**MPV (um^3^)**	0.547 (0.427 – 0.667)	0.443	<8.15	31.1	28.9
**PCT (%)**	0.596 (0478 – 0.714)	0.117	<0.17	42.2	24.4
**PDW (%)**	0.572 (0.453 – 0.691)	0.239	<12.05	44.4	28.9
**PLCR (%)**	0.521 (0.401 – 0.642)	0.726	<11.30	24.4	24.4
**NLR**	**0.823 (0.740 – 0.906)**	<0.001*	>2.56	71.1	22.2
**PLR**	0.572 (0.453 – 0.691)	0.237	<88.41	31.1	24.4
**MLR**	**0.686 (0.576 – 0.796)**	0.002*	>0.18	64.4	28.9

* Statistically significant

## Discussion

Preeclampsia (PE) is associated with a high disease burden with prolong hospital stay, increased maternal mortality and perinatal morbidity.^6^ Early detection using cheap hematological markers of SIR and early intervention could change the dynamics of this disorder and pregnancy outcomes.

Our study demonstrated that patients with PE had a significant increase in total WBC and absolute neutrophil count (ANC). Conversely, there was a significant reduction in absolute lymphocyte count (ALC), and absolute monocyte count (AMC). These findings agreed in part with the study by Lurie et al. which only reported a significant increase in absolute neutrophil count.^17^ However, a different study by Wang et al^9^ reported significant differences in absolute neutrophil count (ANC), absolute lymphocyte count (ALC), and absolute monocyte count (AMC), which are indicative of the inflammatory role of activated leucocytes in maternal circulation in preeclampsia.^2^

RDW has been associated with increased inflammation and oxidative stress.^18^ While our study demonstrated that RDW significantly increases in preeclampsia, it conflicts with reports from a Sudanese study that reported no significant change in RDW in PE and also no correlation with preeclampsia.^12^

Our study demonstrated a significantly higher NLR, significantly lower PLR and a non-significantly higher MLR in preeclampsia compared to normal control. Reports from earlier studies on the relationship of NLR, PLR, and MLR with preeclampsia have shown a lot of inconsistencies. Wang et al., reported that the values of NLR and MLR but not PLR in pre-eclampsia differed significantly from the control.^9^ Increased levels of NLR have also been reported in pre-eclampsia by Oylumlu et al.^19^. These findings are due to the increased release of cellular mediators of inflammation observed in preeclampsia, although the inconsistency observed in the levels of these biomarkers in some studies could be due to race, ethnicity or the testing protocol used.

There was a statistically significant but weak correlation between diastolic blood pressure and NLR and PLR, and weak positive correlation between systolic blood pressure and RDWC (%) for the control group and not for study group. Although it has been established that norma pregnancy evokes a mild systemic inflammatory response, which varies from woman to woman.^2^ It remains unclear why these changes occurred only in normal Pregnancy. Preeclampsia is a multisystem disorder associated with chronic inflammation. There could be other biochemical variables secreted into the circulation that may have altered the natural response of blood pressure to these hematological variables. More robust study design (longitudinal approach) with a larger sample size to ascertain possible cause and effect is advocated.

PLR has shown potential as a marker of SIR in pre-eclampsia in our study. Kim et al., had previously demonstrated that PLR levels differed significantly between preeclampsia and control.^20^ Another study by Yücel & Ustun agreed with our study and reported significantly lower levels of PLR in preeclampsia.^12^ The reports from Banyeh et al., also agreed with our findings of a significant difference in the value of MLR between preeclampsia and controls.^21^ The recruitment of cellular mediators of inflammation; neutrophils, platelets, lymphocytes, and monocytes as a component of the systemic inflammatory response seen in preeclampsia may be the contributing factor.

This study did not show a significantly lower platelet count and platelet indices (PCT, MPV, PDW, PLCR) in patients with pre-eclampsia compared to normotensive controls. However, Tesfay et al,^22^ reported significantly higher MPV in preeclamptic women than in the control group, which disagreed with our findings of lower MPV in preeclamptic women.

Despite the continuous platelet aggregation and destruction of platelets that have been observed and associated with preeclampsia, 23 we found no significant difference in PDW and PLCR between women with preeclampsia and controls in this study. However, some studies have shown a significantly higher level of PDW in women with preeclampsia.^11^ As a result of nonstatistical differences observed in the platelet count and platelet indices, their reliability as markers of SIR cannot be ascertained from our study.

NLR and MLR with a score of (AUC:0.82 and 0.0.69) respectively from this study were considered good and poor predictors of pre-eclampsia. In this study NLR (sensitivity, 71%) is better than MLR (sensitivity 64%) for screening for preeclampsia. Both demonstrated low specificity and as such are poor for the diagnosis of preeclampsia. Our study demonstrated that the AUC of NLR was higher than was earlier reported by Wang et al^9^ but not as high as the AUC reported by a Turkish study.^24^ The sensitivity of NLR was the lowest at 71 % compared to 84.4% by Mannaerts et al. 25 Like NLR, the sensitivity of MLR (64.0%) was also lower than that reported by Wang et al.^9^ When the sensitivity and specificity of NLR are considered together, the study by Oylumlu et al 19 did better than our study. However, the NLR in this study was better at detecting preeclampsia than what was reported by Wang et al.^9^

There is potential for clinical application of these SIR markers toward early detection of preeclampsia. With the high disease burden of preeclampsia especially in resource-poor settings, such cheap and accessible markers of SIR provide the option of quick and early detection of preeclampsia. The study also bridges the knowledge gap on ability of these markers to detect preeclampsia in women generally and in Nigerian women. Yet there are still unanswered questions, the predictors of SIR in preeclampsia, the trend of these variable in longitudinal design through pregnancy and in a larger sample size of study participants

This study drew its strength from being a quick and cheap snapshot assessment of the study subjects and there was no loss to follow ups or dropouts from the study or recollection bias. Another strength of the study was the multiple variable assessment that was done

## Limitations

Limitations of this study are those of cross-sectional design, thus we encourage longitudinal design, randomised controlled trial, systematic reviews and meta-analysis. In addition, predictors of systemic inflammatory response in preeclampsia were not investigated. Although the recommended sample size for preeclampsia was achieved, it may not be representative and, increasing the sample size may allow more robust comparisons to previous studies.

## Conclusions

The RDW, PLR, and NLR with absolute neutrophil, lymphocyte, and monocyte count are significantly different in preeclampsia compared to normotensive pregnant controls. The NLR and MLR are good in detecting preeclampsia, although NLR is better. There may be potential for clinical application of these SIR markers toward early detection and intervention in preeclampsia especially in resource-limited settings like our environment. There is need for robust multi centered larger sample size studies.
